# d-β-Hydroxybutyrate and melatonin for treatment of porcine hemorrhagic shock and injury: a melatonin dose-ranging study

**DOI:** 10.1186/s13104-017-2975-0

**Published:** 2017-11-29

**Authors:** Andrea Wolf, Kristine E. Mulier, Sydne L. Muratore, Gregory J. Beilman

**Affiliations:** Department of Surgery, University of Minnesota, 420 Delaware St SE, Minneapolis, MN 55455 USA

**Keywords:** Blood loss, Resuscitation, Melatonin, Ketone bodies, Antioxidant, Hibernation

## Abstract

**Objective:**

Treatment with a combination of d-β-hydroxybutyrate (BHB) and melatonin (M) improves survival in hemorrhagic shock models. Our objective was to find the most effective melatonin concentration in combination with 4 molar BHB (4 M BHB). Survival and markers of organ injury were analyzed in pigs exposed to pulmonary contusion, liver crush injury, and hemorrhagic shock and treated with lactated Ringer’s solution; 4 M BHB/43 mM M; 4 M BHB/20 mM M; 4 M BHB/10 mM M; 4 M BHB/4.3 mM M; or 4 M BHB/0.43 mM M. This work is an extension of a previously published research study.

**Results:**

Survival was highest in pigs receiving 4 M BHB/43 mM M (13/14), followed by lactated Ringer’s solution (11/16) and BHB/M with decreased melatonin concentrations (4 M BHB/20 mM M 3/6, 4 M BHB/10 mM M 2/6, 4 M BHB/4.3 mM M 3/6, 4 M BHB/0.43 mM M 1/6, p = 0.011). High mortality was associated with increases in serum lactate, higher liver and muscle injury markers and decreases in PaO_2_:FiO_2_ ratios. Our study indicates that treatment with 4 M BHB and melatonin concentrations below 43 mM lack the survival benefit observed from 4 M BHB/43 mM melatonin in pigs experiencing hemorrhagic shock and polytrauma.

**Electronic supplementary material:**

The online version of this article (10.1186/s13104-017-2975-0) contains supplementary material, which is available to authorized users.

## Introduction

Hemorrhagic shock, the state induced by severe blood loss, is the second leading cause of injury-related death [1]. Many deaths occur during the first hour after injury, often before bleeding is adequately controlled [2, 3]. In addition to bleeding control, patients receive resuscitation fluids to restore intravascular volume and tissue perfusion, however, the optimal resuscitation fluid and protocol has not been identified [4, 5]. It has been recognized that currently used resuscitation fluids can have adverse effects themselves [6, 7]. Hence, there is significant need for novel treatments for the early phase of hemorrhagic shock. Infusion of a combination of 4 M d-β-hydroxybutyrate/43 mM melatonin (BHB/M) during early hemorrhagic shock significantly decreased mortality in preclinical rat and pig models [8, 9]. The treatment was developed after the observation that levels of d-β-hydroxybutyrate (BHB), a ketone body, and melatonin (M), an antioxidant, increase in hibernators during torpor and arousal, respectively [10–13].

The goal of this study was to establish the melatonin concentration that in combination with 4 M BHB most effectively improves post-shock survival. We tested the effects of decreased melatonin concentrations in BHB/M in our established porcine hemorrhagic shock, trauma and resuscitation model [14]. We focused on melatonin, as preceding experiments showed that in rat hemorrhagic shock, the concentration of melatonin, but not BHB in the treatment could be decreased without loss of efficacy [15]. We hypothesized that solutions containing 4 M BHB and a melatonin concentration of 43 mM would be equally as effective at improving post-hemorrhagic shock survival as solutions with 4 M BHB in combination with 20, 10, 4.3, or 0.43 mM melatonin.

## Main text

### Methods

#### Shock, treatment and resuscitation

**Fig. 1:**
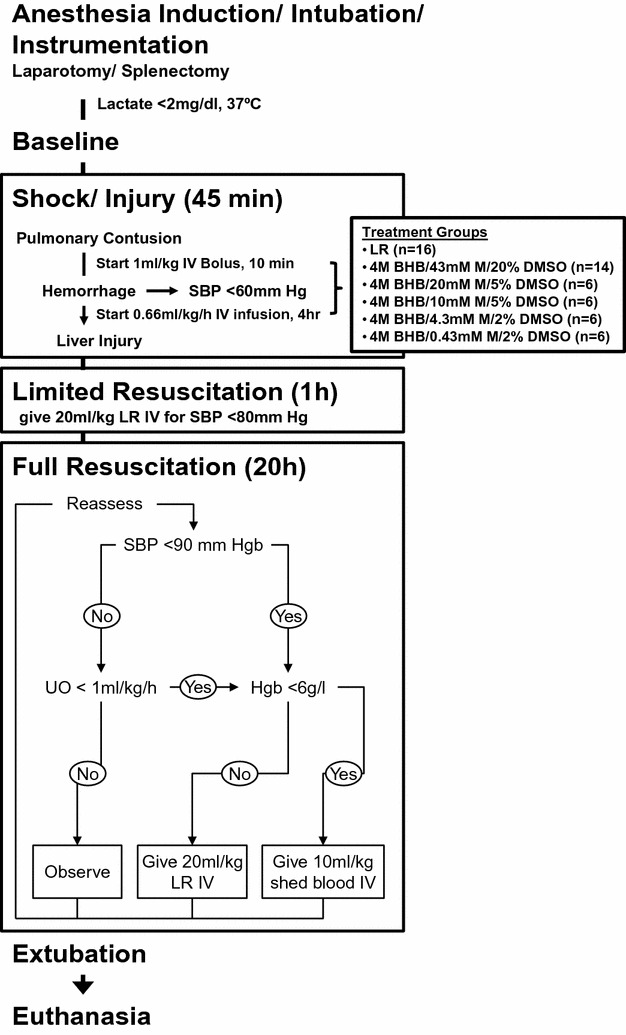
Shock, injury and resuscitation protocol. Pulmonary contusion was followed by blood withdrawal and creation of liver crush injuries with a Holcomb clamp [35]. Fifteen minutes after contusion, treatment solutions were administered as a 1 ml/kg bolus, immediately followed by 0.66 ml/kg/h continuous infusion over 4 h (3.64 ml total). Pigs received limited resuscitation, throughout which they were evaluated every 10 min and, if necessary, received boluses of lactated Ringer’s solution (LR). An hour later, full resuscitation was initiated, throughout pigs received intravenous LR and shed blood. *BHB* d-β-hydroxybutyrate, *DMSO* dimethyl sulfoxide, *Hgb* hemoglobin, *IV* intravenous, *LR* lactated Ringer’s solution, *M* melatonin, *SBP* systolic blood pressure, *UO* urine output (Modified from [14])

All procedures were approved by the University of Minnesota Institutional Animal Care and Use Committee (Protocol # 1306-30703A) and in accordance with the National Institutes of Health guidelines for ethical animal research. Fifty-four (28 male, 26 female) Yorkshire-Landrace pigs (15–25 kg, Manthei Hog Farm, LLC, Elk River, Minnesota) were exposed to our established shock and injury protocol (Fig. 1). Induction of anesthesia, instrumentation, shock and injury, treatment infusion, limited resuscitation (R) and full resuscitation (FR), hemodynamic measurements, and analysis of blood gases, organ function markers and drug serum levels have been previously described in detail [14].

Surviving animals were extubated and recovered for 24 h, 48 h or 14 days. Pigs received ceftiofur (5 mg/kg intravenous daily), analgesia was administered during anesthesia [buprenorphine (0.03 mg/kg subcutaneous every 4 h)] and after arousal [ketoprofen (2 mg/kg daily), buprenorphine (0.03 mg/kg twice daily)]. Animal care staff performed postoperative checks on wellness, body temperature, respiration and pulse at least twice daily. Pigs experiencing unrelieved pain or stress during recovery were sacrificed. Euthanasia was performed with beuthanasia solution (0.22 ml/kg intravenous).

#### 8-Isoprostane ELISA

For 8-isoprostane analysis, urine samples were centrifuged (1000 RpM, 5 min), the supernatant was mixed 1:1 with 1 M acetate buffer (pH 4), extracted using C-18 columns and quantified with ELISA (Cayman Chemical, Ann Arbor, MI) according to manufacturer’s instructions. 8-Isoprostane levels were normalized to urine output per body weight per hour [16]. Samples collected at FR 7 h were used when material from FR 8 h was not available.

#### Statistical analysis

The presented work is an extension of earlier experiments, and 24 of the pigs analyzed here were part of a previously published study (12 in the lactated Ringer’s (LR), 12 in the 4 M BHB/43 mM melatonin group) [14]. To increase the power of the original study, we increased both original groups. However, to save BHB, a significant cost factor in our study, we opted to add fewer pigs to the 4 M BHB/43 mM M than the LR group. Survival was analyzed via Kaplan–Meier analysis with generalized Wilcoxon test. AUCt was calculated using PKSolver from Baseline over five sampling time points (AUC_0-FR20_) using the trapezoidal rule [17]. Non-longitudinal data were analyzed via Kruskal–Wallis test with Dunn–Bonferroni corrections and are reported as medians with interquartile ranges (IQR). Longitudinal parameters were analyzed via Proc Mixed procedure in SAS Version 9.4 (SAS Institute, Inc., Cary, NC) and are depicted at key time points as least-squared means with 95% confidence intervals (CI). Group (G), Time (T) and group * time interaction (G * T) were modeled as fixed effects. The models used compound symmetry, autoregressive or no covariance structure and the between-within method for degrees of freedom. For parameters with significant interaction effects, differences at individual time points were analyzed by pairwise comparisons with Tukey adjustments.

### Results

#### Shock induction and resuscitation

**Fig. 2:**
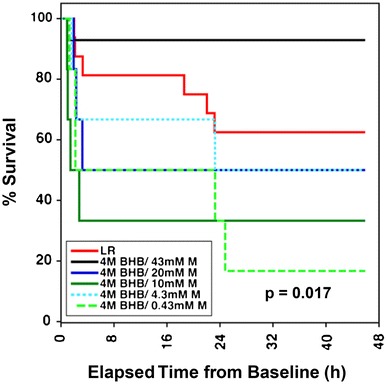
Survival in pigs experiencing hemorrhagic shock and injury. Mean survival in hours [95% CI]: LR 33.1 [24.5, 41.6], 4 M BHB/43 mM M 42.6 [36.5, 48.6], 4 M BHB/20 mM M 24.1 [6.8, 41.4], 4 M BHB/10 mM M 16.3 [0, 33.0], 4 M BHB/4.3 mM M 27.4 [11.7, 43.2], 4 M BHB/0.43 mM M 16.6 [3.5, 29.7]. *BHB* d-β-hydroxybutyrate, *LR* lactated Ringer’s solution, *M* melatonin

There were no significant differences in the amount of blood withdrawn, blood returned or total fluids administered. Pigs treated with 4 M BHB/10 mM melatonin received significantly less LR than those receiving 4 M BHB/0.43 mM melatonin (95% CIs [750, 2206], [2344, 4908] ml/kg, p = 0.029), which is likely due to the high early mortality in this group (Fig. 2).

#### Survival

Twenty-four hours after extubation there was a significant difference in overall survival (p = 0.017, Fig. 2), with the highest rate observed in the 4 M BHB/43 mM melatonin group (13/14), followed by LR pigs (10/16) and those receiving lower doses of melatonin (4 M BHB/20 mM M 3/6, 4 M BHB/10 mM M 2/6, 4 M BHB/4.3 mM M 3/6, 4 M BHB/0.43 mM M 1/6). Survival between 4 M BHB/43 mM M and LR-treated pigs did not differ significantly (p = 0.094). There were significant differences between the 43 mM and the 20, 10 and 0.43 mM melatonin groups (p < 0.05), and between the LR and the 4 M BHB/10 mM melatonin group (p = 0.028).

#### Drug serum levels

BHB/M-treated pigs experienced dose-dependent increases in melatonin and BHB serum concentrations, which peaked at the end of shock and returned to control levels by the end of resuscitation (Additional file 1: Figure S1). Melatonin concentrations in 4 M BHB/43 mM melatonin pigs were significantly higher than in all other groups after shock and during early resuscitation. Differences were significant between pigs infused with 4 M BHB/20 mM melatonin versus those receiving LR, 4 M BHB/4.3 mM melatonin or 4 M BHB/0.43 mM melatonin at the end of shock. At the end of shock, BHB concentrations were higher in all BHB/M groups than in controls. We observed some variability in BHB levels which resulted in significant group differences, however, there was no obvious effect of melatonin dose on BHB serum concentrations. Total drug exposure over time followed the patterns observed for drug serum levels.

#### Hemodynamic physiologic parameters

Key hemodynamic and physiologic parameters are depicted in (Additional file 2: Table S1). Hemorrhage caused a drop in mean arterial pressure and cardiac output along with increases in heart rate in all groups, which recovered during resuscitation. Urine output did not differ significantly between groups at individual time points. BHB/M treatment increased sodium and decreased potassium levels during early resuscitation, a previously described effect that was independent of treatment melatonin concentration [9]. We observed shock-induced decreases in pH which returned towards baseline levels during resuscitation. There were no obvious BHB/M-treatment or melatonin dose-dependent effects on hemoglobin or serum levels of blood urea nitrogen and lactate dehydrogenase. There were no obvious treatment-dependent effects on body temperature, mean pulmonary artery pressure, pulmonary artery occlusion pressure, bladder pressure, mixed venous oxygen saturation, oxygen consumption, serum levels of alanine aminotransferase, albumin, total protein, bilirubin and alkaline phosphatase (not shown).

**Fig. 3:**
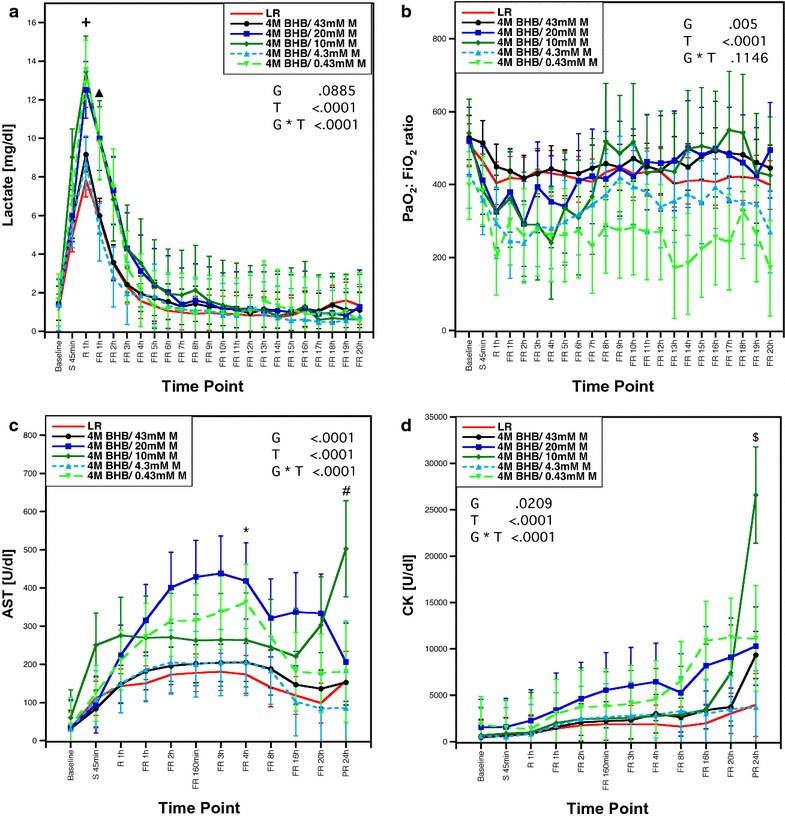
**a** Lactate levels, **b** PaO2:FiO2 ratios, **c** AST levels and **d** creatine kinase concentrations throughout the experiment. Data presented as least-squares means with 95% confidence intervals. ^+^p < 0.05 for LR vs 4 M BHB/10mM M; ^▲^p < 0.05 for LR vs 4 M BHB/20 mM M and 4 M BHB/10 mM M and 4 M BHB/0.43 mM M, 4 M BHB/0.43 mM M vs 4 M BHB/43 mM M and 4 M BHB/4.3 mM M. *p < 0.05 for LR vs 4 M BHB/20 mM M; ^#^ for 4 M BHB/10 mM M versus 4 M BHB/4.3 mM M and 4 M BHB/43 mM M; ^$^ for 4 M BHB/10 mM M vs LR and 4 M BHB/43 mM M and 4 M BHB/20 mM M and 4 M BHB/4.3 mM M. *AST* aspartate aminotransferase, *BHB* d-β-hydroxybutyrate, *CK* total creatine kinase, *FiO*_*2*_ inspired fraction of oxygen, *FR* full resuscitation, *G* group effect, *G* * *T* group * time interaction effect, *LR* lactated Ringer’s solution, *M* melatonin, *PaO*_*2*_ partial arterial pressure of oxygen, *R* limited resuscitation, *S 45* *min* end of shock period, *T* time effect

Lactate levels peaked during limited resuscitation but returned to baseline levels by the end of the experiment (Fig. 3a). BHB/M-treated pigs receiving low melatonin concentrations experienced dose-dependent decreases in PaO_2_:FiO_2_ ratios during early resuscitation (Fig. 3b). Pigs treated with BHB/M experienced increases in serum concentrations of aspartate aminotransferase (AST) and creatine kinase (CK) (Fig. 3b, c). The shock-induced disturbances were most prominent in groups with high early mortality rates, namely in pigs receiving 4 M BHB with 20, 10 and 0.43 mM melatonin.

8-Isoprostane urine levels were analyzed as markers of trauma-induced oxidative stress [18]. There was an insignificant trend towards lower 8-isoprostane levels in the groups treated with BHB/M during resuscitation (Additional file 3: Figure S2). This effect was independent of the melatonin concentration in the treatment.

### Discussion

4 M BHB/43 mM M significantly improves survival in preclinical hemorrhagic shock models [8, 9]. Here, we describe experiments to optimize the melatonin concentration in the treatment. Earlier experiments showed that in pigs exposed to hemorrhagic shock and injury, doubling the dose of 4 M BHB/43 mM M resulted in increased mortality (unpublished data). In rats, lowering the BHB concentration in combination with 43 mM M resulted in a trend towards decreased survival, while survival times were retained when melatonin levels were lowered [15]. Based on these results, we used our porcine hemorrhage, injury and resuscitation model to evaluate treatment solutions containing 4 M BHB in combination with 0.43–43 mM melatonin. We hypothesized that the melatonin concentration could be decreased without loss of efficacy.

Mortality in pigs receiving BHB/M containing below-standard melatonin concentrations exceeded that in the control group. This was surprising, as previous studies suggest a beneficial effect of BHB in hemorrhagic shock in both rats and pigs, acting synergistically with melatonin. In rats experiencing hemorrhagic shock, 4 M BHB alone significantly improved survival, and lowering the BHB concentration in BHB/M was associated with a trend towards increased mortality [8, 15].

Our data suggests that treatment with BHB/M containing decreased melatonin levels increased shock-induced lung and organ injury. These animals experienced increased lactate levels and elevated serum concentrations of AST and CK, markers of liver and muscle injury. Pulmonary contusion and hemorrhagic shock induce pulmonary inflammation, which can lead to hypoxemia and acute respiratory distress syndrome [19, 20]. We observed melatonin dose-dependent decreases in PaO_2_:FiO_2_ ratios during early resuscitation, indicating increased lung injury. This was unexpected, as both melatonin and BHB exhibit anti-inflammatory effects and BHB decreases inflammation and apoptosis in rats and pigs exposed to severe blood loss [21–30]. However, melatonin effects in hemorrhagic shock can be dose-dependent, while ketone bodies may be pro-inflammatory at high doses [31–34]. With systemic melatonin levels insufficient to counteract the effects of shock and injury, the high BHB dose may have exacerbated trauma-induced inflammation. This was not associated with increased oxidative stress, as we observed a trend towards decreased 8-isoprostane levels in BHB/M treated pigs, which was independent of the melatonin concentration in the treatment.

### Conclusions

Hemorrhagic shock is the leading cause of preventable death after injury, with many of these deaths occurring in the prehospital phase. 4 M BHB/43 mM M is a low-volume resuscitation fluid that significantly improves survival when administered during early hemorrhage in preclinical models of hemorrhagic shock and injury [8, 9, 15]. Optimization of treatment dose is an important step towards translation from preclinical to clinical use. Here, we tested the efficacy of 4 M BHB in combination with 0.43–43 mM melatonin in porcine hemorrhagic shock, injury and resuscitation. Treatment with below-standard melatonin concentrations resulted in mortality rates exceeding that in the control group. Lowered melatonin treatment concentrations resulted in increased markers of lung, liver and kidney injury, suggesting that decreased melatonin serum levels were insufficient to counteract BHB-induced increases in inflammation. Our research underlines the importance of reaching adequate systemic BHB and melatonin levels, while illustrating the narrow therapeutic window of the treatment in its current formulation.

## Limitations

This study has several limitations. As the melatonin dose-ranging experiments were an extension of our previously published study, we expanded the original 4 M BHB/43 mM M and LR groups and added treatments to our original experiment [14]. Consequently, group sizes were uneven and animals were not completely randomized, rendering a risk for model variation over time. However, BHB/M at the standard dose exerted a robust beneficial effect and consistently outperformed LR in our model (2/2 additional BHB/M pigs survived, while only 1/4 of LR pigs survived).

As we did not include dimethyl sulfoxide (DMSO)-treated control groups in our experiments, it is unclear whether changes in DMSO concentrations affected efficacy in the low-dose melatonin groups. Previously, BHB/M- treatment was significantly more effective at increasing post-shock survival than treatment with isosmotic solutions containing equal DMSO concentrations [9]. As we concluded that it was unlikely that changes in DMSO concentration affected survival, we opted for LR as control to represent the standard of care.

Survival differences between 4 M BHB/43 mM M and the control group were not significant in this study (Fig. 2), which is likely due to the limited sample size used. As our experiments clearly showed that decreasing melatonin concentrations was detrimental, we limited our sample size to save animals and resources.

## Additional files

**Additional file 1.** Average drug serum levels (a, b) and drug exposure over time (c, d) during hemorrhagic shock and injury.

**Additional file 2.** Physiologic parameters and markers of organ function in pigs exposed to hemorrhagic shock, injury and resuscitation.

**Additional file 3.** Urine 8-isoprostane levels during hemorrhagic shock and injury.

## Abbreviations

AST: aspartate aminotransferase
AUC: area under the curve
BHB: d-β-hydroxybutyrate
CK: total creatinine kinase
DMSO: dimethyl sulfoxide
FiO_2_: inspired fraction of oxygen
FR: full resuscitation
G: group effect
G * T: group * time interaction effect
Hgb: hemoglobin
IQR: interquartile range
IV: intravenous
LR: lactated Ringer’s solution
M: melatonin
PaO_2_: partial arterial pressure of oxygen
PR: post resuscitation
R: limited resuscitation
SBP: systolic blood pressure
SvO_2_: mixed venous oxygen saturation
T: time effect
UO: urine output
