# Supplementary material for: d-β-Hydroxybutyrate and melatonin for treatment of porcine hemorrhagic shock and injury: a melatonin dose-ranging study
Source: BMC Res Notes. 2017 Nov 29;10:649. doi: 10.1186/s13104-017-2975-0 (PMC5707828; doi:10.1186/s13104-017-2975-0)
Supplement: Supplementary file 1 — Additional file 1. Average drug serum levels (a, b) and drug exposure over time (c, d) during hemorrhagic shock and injury. [file 13104_2017_2975_MOESM1_ESM.pdf]

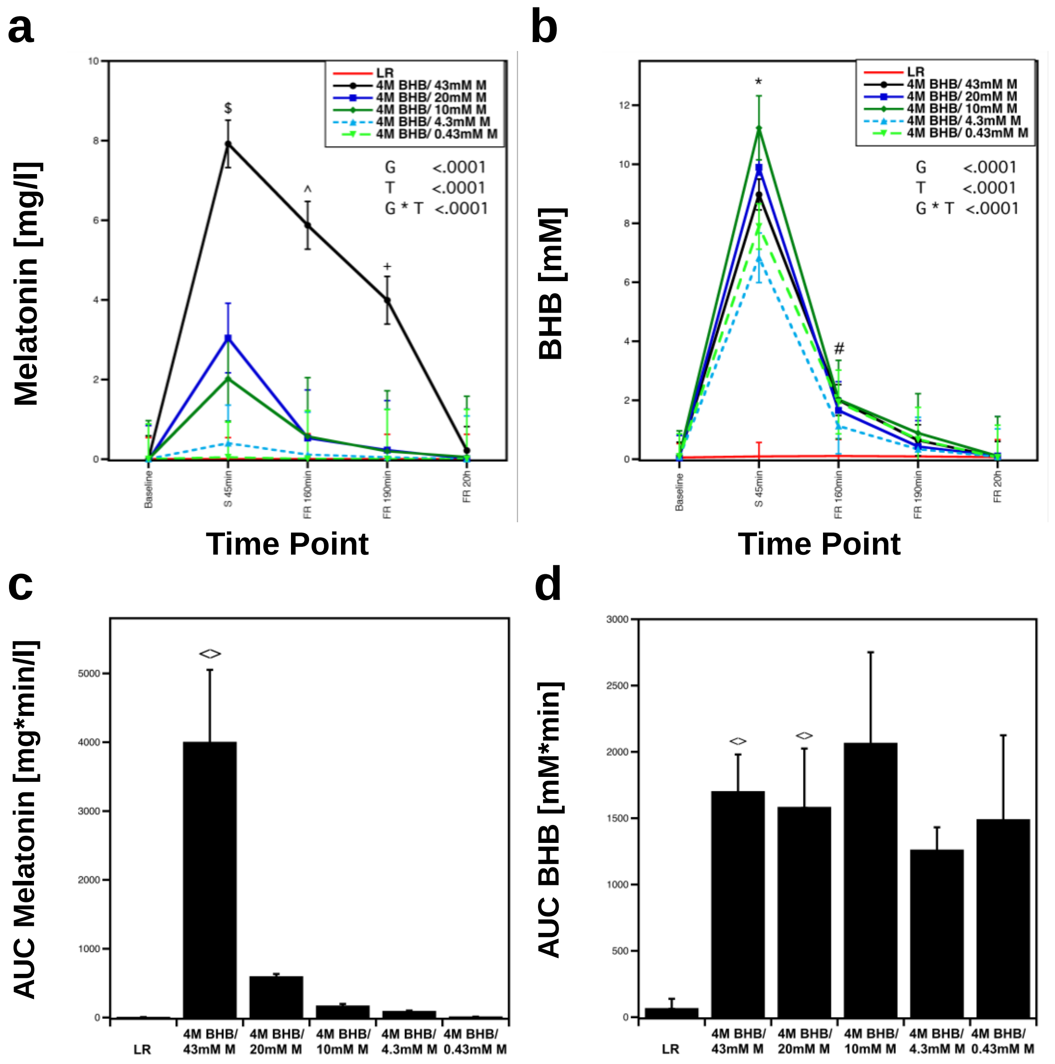

Supplemental Figure 1. Average drug serum levels (a,b) and drug exposure over time (c,d) during hemorrhagic shock and injury. Data presented as least-squares means with 95% confidence intervals (a, b) or as medians with IQR (c-f). AUCt Melatonin in mg*min/l [95% CI]: LR [7, 7], 4M BHB/ 43mM M [1418, 6852], 4M BHB/ 20mM M [411, 633], 4M BHB/ 10mM M [145, 198], 4M BHB/ 4.3mM M [73, 108], 4M BHB/ 0.43mM M [11, 13]. AUCt BHB in mM*min [95% CI]: LR [25, 184], 4M BHB/ 43mM M [1026, 3586], 4M BHB/ 20mM M [1578, 2025], 4M BHB/ 10mM M [1383, 2751], 4M BHB/ 4.3mM M [888, 1454], 4M BHB/ 0.43mM M [1169, 2126]. $ p<0.05 for 4M BHB/43mM M vs all other treatments and 4M BHB/20mM M vs LR, 4M BHB/4.3mM M and 4M BHB/0.43mM M; ^ p<0.05 for 4M BHB/43mM M vs all other treatments; + p<0.05 for 4M BHB/43mM M vs all other treatments; * p<0.0.5 for LR vs all other treatments, 4M BHB/4.3mM M vs 4M BHB/43mM M and 4M BHB/20mM M and 4M BHB/10mM M, 4M BHB/10mM M vs 4M BHB/0.43mM M; # p<0.05 for LR vs 4M BHB/43mM M; <> p<0.05 vs LR. AUC- area under the curve, BHB - D-β-hydroxybutyrate, FR – full resuscitation, G - Group effect, G*T - Group * Time interaction effect, LR - lactated Ringer's solution, M - melatonin, S 45 min end of shock period. T - Time effect.
