# Supplementary material for: d-β-Hydroxybutyrate and melatonin for treatment of porcine hemorrhagic shock and injury: a melatonin dose-ranging study
Source: BMC Res Notes. 2017 Nov 29;10:649. doi: 10.1186/s13104-017-2975-0 (PMC5707828; doi:10.1186/s13104-017-2975-0)
Supplement: Supplementary file 2 — Additional file 2. Physiologic parameters and markers of organ function in pigs exposed to hemorrhagic shock, injury and resuscitation. [file 13104_2017_2975_MOESM2_ESM.pdf]

Parameter	Baseline	S 45min	R 1hr	FR 2hr	FR 20hr	PR 24hr	Effects
**Mean Arterial Pressure [mm Hg]**							
LR	88 (82-94)	41 (35-47)	58 (52-64)	74 (67-80)	68 (61-76)		G 0.0071
4M BHB/43mM M	85 (78-91)	41 (34-47)	59 (52-65)	73 (66-80)	72 (65-79)		T <.0001
4M BHB/20mM M	80 (70-90)	40 (31-50)	44 (33-55)	66 (52-79)	67 (53-81)		G*T 0.0091
4M BHB/10mM M	92 (82-102)	35 (23-46)	62 (47-76)	64 (48-81)	96 (79-113)		
4M BHB/4.3mM M	88 (78-98)	34 (24-43)	48 (38-58)	65 (53-76)	66 (54-78)		
4M BHB/0.43mM M	95 (85-105)	44 (34-54)	48 (37-59)	67 (52-82)	59 (45-73)		
**Heart rate [beats/ min]**							
LR	120 (104-136)	237 (222-253)	175 (159-192)	169 (152-186)	158 (139-177)		G 0.0002
4M BHB/43mM M	125 (108-142)	198 (181-215)	179 (161-196)	151 (133-168)	144 (126-161)		T <.0001
4M BHB/20mM M	137 (111-163)	254 (228-279)	205 (176-234)	217 (182-253)	146 (110-183)		G*T 0.0015
4M BHB/10mM M	135 (109-161)	250 (220-279)	197 (158-237)	186 (142-230)	153 (108-198)		
4M BHB/4.3mM M	144 (118-170)	218 (193-244)	147 (120-174)	153 (122-184)	126 (94-158)		
4M BHB/0.43mM M	151 (125-177)	264 (239-290)	189 (160-219)	198 (158-239)	166 (130-202)		
**Cardiac Output [l/min]**							
LR	2.9 (2.4-3.4)	1.3 (0.9-1.8)	2.9 (2.4-3.5)	4.4 (3.9-5.0)	4.3 (3.7-4.9)		G 0.0157
4M BHB/43mM M	2.9 (2.4-3.4)	1.7 (1.1-2.3)	4.1 (3.6-4.7)	5.1 (4.5-5.6)	4.0 (3.5-4.6)		T <.0001
4M BHB/20mM M	3.0 (2.3-3.8)	1.5 (0.7-2.3)	2.3 (1.3-3.3)	4.2 (3.1-5.3)	3.2 (2.1-4.3)		G*T 0.1389
4M BHB/10mM M	3.2 (2.4-3.9)	0.9 (0.1-1.8)	2.3 (1.1-3.5)	3.6 (2.3-5.0)	3.8 (2.5-5.1)		
4M BHB/4.3mM M	3.3 (2.5-4.1)	1.4 (0.6-2.1)	3.6 (2.7-4.5)	4.6 (6.7-5.6)	3.4 (2.4-4.3)		
4M BHB/0.43mM M	4.0 (3.2-4.7)	1.2 (0.4-1.9)	2.5 (1.5-3.5)	5.3 (4.0-6.6)	4.5 (3.2-5.7)		
**Urine Output [ml/hr]**							
LR	39 (-10-88)	18 (-31-66)	71 (20-122)	87 (35-140)	107 (47-168)		G 0.0625
4M BHB/43mM M	63 (11-114)	17 (-36-69)	132 (79-185)	152 (99-206)	140 (87-194)		T <.0001
4M BHB/20mM M	20 (-58-99)	18 (-61-97)	37 (-52-127)	72 (-36-180)	41 (-71-152)		G*T 0.0911
4M BHB/10mM M	92 (14-171)	5 (84-95)	45 (-74-164)	75 (-58-209)	89 (-47-226)		

4M BHB/4.3mM M	51 (-28-129)	17 (-62-95)	155 (72-238)	75 (-19-170)	120 (24-216)		
4M BHB/0.43mM M	67 (-12-145)	22 (-56-101)	38 (-51-128)	200 (73-327)	84 (-27-195)		
**Na^+ [mEq/l]**							
LR	139.0 (136.7-141.4)	138.4 (136.0-140.7)	139.5 (136.9-142.0)	137.7 (135.1-140.3)	142.1 (139.2-145.1)		G <.0001
4M BHB/43mM M	139.2 (136.7-141.7)	143.8 (141.2-146.4)	145.6 (143.0-148.2)	148.2 (146.5-150.0)	142.9 (140.3-145.5)		T <.0001
4M BHB/20mM M	140.5 (136.7-144.3)	145.2 (141.3-149.0)	147.7 (143.2-152.3)	150.2 (144.8-155.6)	144.0 (138.6-149.4)		G*T 0.4134
4M BHB/10mM M	140.5 (136.7-144.3)	144.3 (139.7-148.8)	150.5 (144.2-156.9)	149.3 (142.7-156.0)	152.5 (145.9-159.1)		
4M BHB/4.3mM M	142.8 (139.0-146.7)	144.3 (140.5-148.2)	146.8 (142.7-151.0)	148.9 (144.2-153.6)	146.0 (141.3-150.7)		
4M BHB/0.43mM M	140.7 (136.8-144.5)	145.0 (141.2-148.8)	148.1 (143.0-153.2)	149.8 (143.3-156.4)	144.3 (138.9-149.8)		
**K^+ [mEq/l]**							
LR	3.9 (3.7-4.2)	5.8 (5.6-6.1) #	3.9 (3.7-4.2) #	4.3 (4.1-4.6) #	3.3 (3.0-3.6)		G <.0001
4M BHB/43mM M	4.0 (3.7-4.2)	4.6 (4.3-4.8) *	3.0 (2.7-3.3) *△	3.2 (2.9-3.4) *○	3.3 (3.0-3.5)		T <.0001
4M BHB/20mM M	3.8 (3.4-4.2)	5.0 (4.6-5.4)	4.4 (4.0-4.9) #	3.6 (3.0-4.1)	3.0 (2.5-3.6)		G*T <.0001
4M BHB/10mM M	3.8 (3.4-4.2)	5.5 (5.0-6.0)	3.1 (2.4-3.7)	3.2 (2.6-3.9)	2.6 (1.9-3.3)		
4M BHB/4.3mM M	3.8 (3.4-4.2)	5.0 (4.6-5.4)	3.0 (2.6-3.4)	3.0 (2.6-3.5) *	3.5 (3.0-3.9)		
4M BHB/0.43mM M	4.0 (3.6-4.4)	5.7 (5.3-6.0)	3.5 (3.0-4.1)	3.0 (2.4-3.7)	3.0 (2.5-3.6)		
**pH**							
LR	7.47 (7.43-7.50)	7.39 (7.35-7.42)	7.28 (7.25-7.32) ○●	7.38 (7.34-7.41) #	7.42 (7.38-7.46)		G <.0001
4M BHB/43mM M	7.45 (7.42-7.49)	7.44 (7.40-7.47)	7.38 (7.34-7.41) ●	7.50 (7.47-7.54) *	7.48 (7.44-7.51)		T <.0001
4M BHB/20mM M	7.46 (7.40-7.51)	7.39 (7.33-7.45)	7.25 (7.18-7.31)	7.36 (7.29-7.44)	7.49 (7.41-7.57)		G*T <.0001
4M BHB/10mM M	7.47 (7.41-7.53)	7.39 (7.33-7.45)	7.28 (7.20-7.36)	7.43 (7.34-7.53)	7.45 (7.36-7.55)		
4M BHB/4.3mM M	7.48 (7.42-7.53)	7.44 (7.39-7.50)	7.44 (7.38-7.50) *●	7.51 (7.45-7.58)	7.39 (7.32-7.46)		
4M BHB/0.43mM M	7.45 (7.39-7.51)	7.43 (7.37-7.48)	7.07 (7.01-7.14) *#○	7.29 (7.20-7.37)	7.36 (7.28-7.43)		

**Hgb [g/dl]**							
LR	8.7 (8.4-9.0)	7.0 (6.6-7.3)	5.2 (4.8-5.5)	6.1 (5.7-6.4)	5.7 (5.3-6.1)		G 0.014
4M BHB/43mM M	8.6 (8.2-8.9)	6.1 (5.8-6.4)	5.0 (4.6-5.3)	6.0 (5.7-6.4)	5.6 (5.3-6.0)		T <.0001
4M BHB/20mM M	8.6 (8.0-9.1)	6.3 (5.8-6.8)	5.6 (5.0-6.2)	6.4 (5.7-7.1)	6.1 (5.4-6.8)		G*T 0.1014
4M BHB/10mM M	8.7 (8.1-9.2)	5.9 (5.3-6.5)	5.3 (4.5-6.1)	6.5 (5.6-7.4)	6.7 (5.8-7.5)		
4M BHB/4.3mM M	8.9 (8.3-9.4)	6.5 (5.9-7.0)	5.2 (4.5-5.9)	6.1 (5.4-6.7)	6.0 (5.3-6.6)		
4M BHB/0.43mM M	8.6 (8.0-9.1)	6.9 (6.4-7.4)	4.5 (3.7-5.2)	5.9 (5.1-6.7)	5.7 (5.0-6.4)		
**Blood urea nitrogen [mg/dl]**							
LR	13.6 (0.6-26.6)	11.3 (-2.1-24.8)	10.6 (-2.8-24.0)	12.8 (-2.2-27.9)	31.5 (15.8-47.2)	16.0 (-20.8-52.8) △	G 0.0095
4M BHB/43mM M	9.5 (-4.4-23.4)	11.9 (-2.5-26.4)	10.8 (-4.3-25.8)	12.6 (-1.8-27.1)	14.4 (-0.1-28.8)	13.0 (-13.0-39) △	T 0.0992
4M BHB/20mM M	8.8 (-12.4-30.1)	11.2 (-12.1-34.5)	12.3 (-13.8-38.3)	15.0 (-15.1-45.1)	17.7 (-12.4-47.7)	207.0 (176.9-237.1) *#▲○●	G*T <.0001
4M BHB/10mM M	9.8 (-11.4-31.1)	11.8 (-14.3-37.8)	8.0 (-28.8-44.8)	9.0 (-27.8-45.8)	12.5 (-24.3-49.3)	8.0 (-28.8-44.8) △	
4M BHB/4.3mM M	8.3 (-12.9-29.6)	11.6 (-11.7-34.9)	9.6 (-13.7-32.9)	11.8 (-14.3-37.8)	11.8 (-14.3-37.8)	16.0 (-14.1-46.1) △	
4M BHB/0.43mM M	9.5 (-11.8-30.8)	11.7 (9.6-32.9)	8.5 (-17.5-34.5)	10.7 (-19.4-40.7)	11.3 (-18.7-41.4)	11.0 (-41.1-63.1) △	
**Lactate dehydrogenase [U/dl]**							
LR	1122 (518-1725)	1320 (715-1924)	1406 (801-2011)	1882 (1267-2497)	2801 (2132-3469) ●	3279 (2524-4035) ●	G 0.0005
4M BHB/43mM M	1098 (453-1744)	1168 (521-1816)	1445 (795-2095)	2431 (1779-3083)	3065 (2399-3731) ●	4563 (3876-5259) △	T <.0001
4M BHB/20mM M	560 (-426-1546)	589 (-401-1580)	908 (-97-1913)	1296 (226-2366)	1480 (153-2807) ●	1307 (-25-2639) #●	G*T <.0001
4M BHB/10mM M	501 (-485-1487)	638 (-384-1660)	854 (-234-1943)	877 (-366-2120)	1457 (-148-3062) ●	1294 (-385-2973) ●	
4M BHB/4.3mM M	1266 (280-2252)	1630 (637-2623)	1659 (659-2659)	2630 (1597-3662)	2863 (1693-4033) ●	3579 (2391-4767) ●	
4M BHB/0.43mM M	1452 (465-2438)	1551 (565-2537)	1983 (978-2987)	3170 (2101-4239)	7857 (6530-9184) *#△▲○	7681 (6244-9118) *△▲○	

Supplemental Table. Physiologic Parameters and Markers of Organ Function in Pigs Exposed to Hemorrhagic Shock, Injury and Resuscitation. Data are presented as least-squared means (95% confidence interval). * p<0.05 vs LR, # p<0.05 vs 4M BHB/43mM M, △ p<0.05 vs 4M BHB/20mM M, ▲ p<0.05 vs 4M BHB/10mM M , ○ p<0.05 vs 4M BHB/4.3mM M , ● p<0.05 vs 4M BHB/0.43mM M. BHB - D- β –hydroxybutyrate, BUN – blood urea nitrogen, G - group effect, G*T - group * time interaction effect, FR - full resuscitation, LDH – lactate dehydrogenase, LR lactated Ringer’s solution, M – melatonin, PR - post resuscitation, R - limited resuscitation, S 45min – end of shock period, T - time effect.
