# Supplementary material for: d-β-Hydroxybutyrate and melatonin for treatment of porcine hemorrhagic shock and injury: a melatonin dose-ranging study
Source: BMC Res Notes. 2017 Nov 29;10:649. doi: 10.1186/s13104-017-2975-0 (PMC5707828; doi:10.1186/s13104-017-2975-0)
Supplement: Supplementary file 3 — Additional file 3. Urine 8-isoprostane levels during hemorrhagic shock and injury. [file 13104_2017_2975_MOESM3_ESM.pdf]

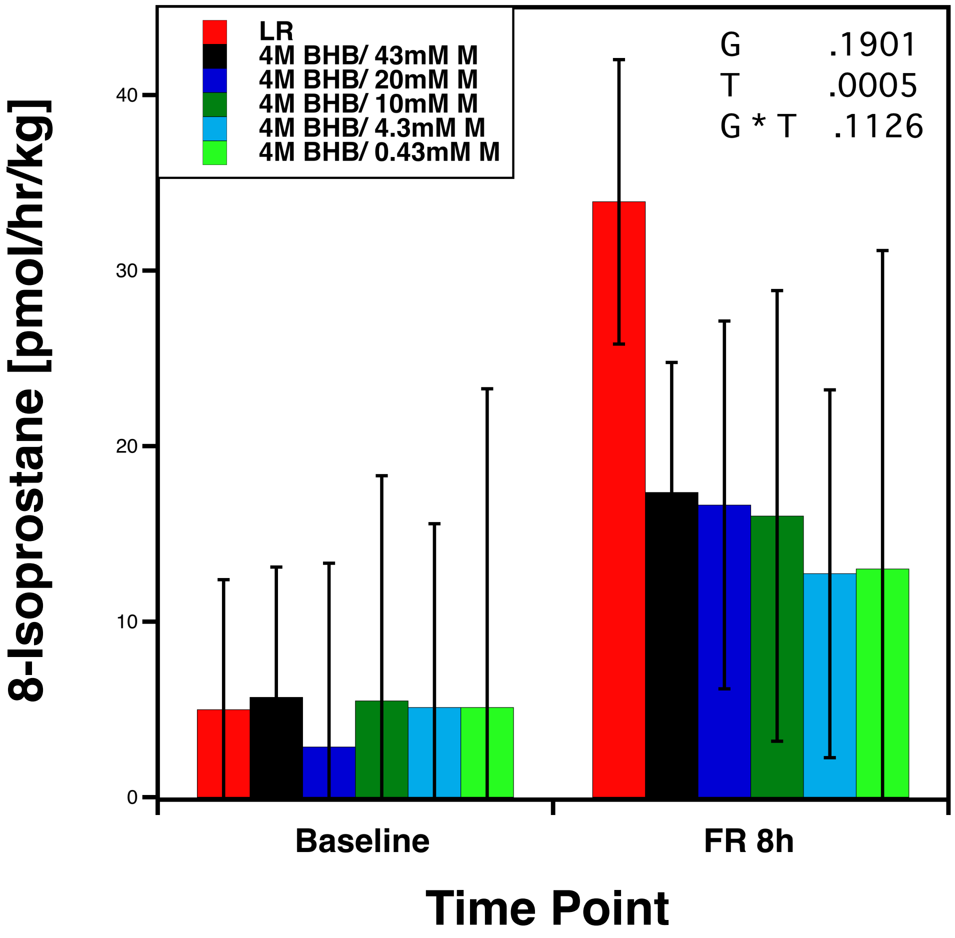

Supplemental Figure 2. Urine 8-Isoprostane levels during hemorrhagic shock and injury. Data are presented as least-squares means with 95% confidence intervals. BHB - D-β–hydroxybutyrate, FR - full resuscitation, G - Group effect, G*T - Group * Time interaction effect, LR - lactated Ringer's solution, M - melatonin, T - Time effect.
